# Epidemiological Pattern of Hepatitis B and Hepatitis C as Etiological Agents for Hepatocellular Carcinoma in Iran and Worldwide

**DOI:** 10.5812/hepatmon.6894

**Published:** 2012-10-24

**Authors:** Ahmed Zidan, Hubert Scheuerlein, Silke Schüle, Utz Settmacher, Falk Rauchfuss

**Affiliations:** 1 Department of General, Visceral and Vascular Surgery, Jena University Hospital, Jena, Germany

**Keywords:** Hepatitis B Virus, Hepatitis C, Iran

## Abstract

**Context:**

Hepatitis B virus (HBV) and hepatitis C virus (HCV) infections constitute a major global health problem. About 60,000 and 350,000 deaths occur as the results of HBV and HCV infections, respectively. Chronic hepatitis B and C infections are leading causes of cirrhosis and hepatocellular carcinoma (HCC) which are considered as the third cancer-associated cause of deaths worldwide. Iran suffers from the same problem but to a lesser extent as it is considered as a low endemic area for HBV and HCV infections and also as a low incidence area of HCC. This study was conducted to assess and provide a clear picture about epidemiology of HBV and HCV infections in Iran and worldwide, with the consequence on HCC distribution all over the world including Iran, and to analyze current literature regarding the modes of transmission and risk factors of HBV and HCV infections.

**Evidence Acquisition:**

In this review, we performed electronic and manual searches on available databases such as MEDLINE, PubMed, Ovid, Embase, and the Iranian databases such as IranMedex. We also performed a Google search to find related articles.

**Results:**

HBV and HCV infections are the most common risk factors of hepatocellular carcinoma. The epidemiology of HCC usually follows that of HBV and HCV infections. With the introduction of HBV national vaccine in Iran and worldwide, there is a noticeable effect on reduction in HBV prevalence in most countries, and we expect that HCV will replace HBV as a major risk factor of HCC in Iran and worldwide. Alcohol plays a minor role as a risk factor for cirrhosis and HCC in Iran, Asia, and Africa, despite its noticeable role in Europe and the USA.

**Conclusions:**

Vaccination against HBV remains the most effective approach against HBV infection with consequence decrease in HBV-related HCC. There is a need to improve the awareness about epidemiology of HBV and HCV infections, modes of transmission, and their complications, specifically HCC among population.

## 1. Context

Hepatitis B virus (HBV) and hepatitis C virus (HCV) infections constitute a major global health problem. About 60,000 and 350,000 deaths occur as the results of HBV and HCV infections, respectively (1). More than 350 million people across the world are infected with HBV and about 170 million with HCV (2). Iran is among those countries with low frequency of HBV and HCV infections (3, 4). Hepatitis B and C are the major risk factors for cirrhosis and hepatocellular carcinoma all over the world. Hepatocellular carcinoma (HCC), which accounts for 70-90% of primary liver cancers (5-7), is the third leading cause of cancer- related deaths worldwide, and its burden is expected to increase further in coming years (6, 8, 9). HCC is the fifth most frequent cancer throughout the world among men with estimated 442,000 new cases per year. Among women, HCC considered as the eighth most frequent cancer in world, with an estimated 184,000 new cases per year (10, 11).

## 2. Evidence Acquisition

We performed electronic and manual searches of the available databases such as MEDLINE, PubMed, Ovid, Embase, and the Iranian databases such as IranMedex; also Google search was performed based on keywords given. Manual search was necessary because not all published data were available online. Data search and collection were done by the first and last authors, and the articles were assessed and criticized by multiple meetings of all authors. We included studies in English from 1980-2012. Author’s name or journal name did not influence the decision for including or excluding the articles.

### 2.1. Hepatitis B and C in Iran

Although most countries in the Middle East region are still considered as intermediate or high endemic areas for hepatitis B infection (12), Iran is among those countries with low frequency of hepatitis B infection (13). It is estimated that approximately 3% of Iranians are chronically infected with hepatitis B virus (14). Reviewing literatures on the prevalence of HBV infection in Iran, it seems that there is a wide epidemiologic difference between different provinces. Iran is a large country with an area of approximately 1,700,000 square Km and the people are living in different parts and provinces of the country with different lifestyles, habits, and ethnicities. So it is not appropriate to compare the results of studies performed in different provinces of Iran. HBV seroprevalence in Iran is asymmetrical and mosaic in nature ranging from 1.7 to 8.9 % in different parts throughout the country, including 1.7% in Fars province (southern region), 1.7% in Tehran province (central region), 5% in Sistan-and-Balouchestan province (eastern region), 2.3% in Hamedan province (western region), and 8.9% in Golestan province (northern region) (15, 16). Alavian et al. described male sex, marital status (being married), extramarital sexual activity, contact with hepatitis B infected subjects, intravenous drug abuse, major surgery, visit to experimental dentists, and some professions (such as policemen, barbers, and drivers) as possible transmission risk factors of hepatitis B infection in Iran (12, 15). The most common routes of HBV infection transmission in Iran are perinatal transmission and intravenous drug abuse (17). The national vaccination program for HBV infection has been started and routinely performed for newborn and high-risk groups since 1992 in Iran; it has changed the epidemiological pattern among the population from vertical to horizontal route. The adulthood vaccination has been introduced in 2007 (18). An epidemiologic survey conducted by Zali et al. before and after the national vaccination program in Iran showed that there was no significant decline in the overall rate of seropositivity, but in the age group of 2-14 years old this rate decreased significantly (1.3% vs. 0.8%, P < 0.05) (15). Thus, the age factor has an important influencing role upon the rates reported by various studies with a noticeable decrease in the prevalence among children and teenagers after the vaccination program was implemented (16); the vaccination program needs decades to decrease total prevalence of hepatitis B infection in Iran. HCV prevalence is affected greatly by the sex factor; men are tenfold more infected than women are (3). This may be due to the fact that HBV transmission in Iran is mainly vertical, from mother to baby with no sexual preference. On the other hand, the transmission through intravenous route or sexual promiscuity is more common among men (19). High-risk populations for HCV infections are those who are in possible activities with contact to infected blood as in blood transfusions, addicts, hemodialysis, tattooing, prisoners, and during medical and dental care (20). Merat et al. performed the first population–based study in Iran to determine the prevalence of HCV infection in the general population. In this study, he avoided selection bias as the target was the general population in three big provinces in Iran; Golestan, Tehran, and Hormozogan. The seroprevalence of HCV in the study population was 0.5%, which was higher than that in previous estimates for blood donors (3) (Table 1). With the gradual decline in the prevalence of HBV infection after the implementation of national vaccination program, HCV infection will replace HBV infection as a major cause of chronic viral liver disease in the next decades. It will be enhanced by the absence of vaccination against HCV up to now and expensive treatment which adds more loads on the medical care system (21-23).

**Table 1:** HCV Prevalence in Iran in Different Studies with Different Target Groups

Author	Year	Target Group	No.	Prevalence
**Alavian *et al.* (85)**	2003	Haemodialysis patients	838	13.2%
**Alizadeh *et al.* (86)**	2005	Drug abuser prisoners (Hamedan)	427	30%
**Zakizad* et al. *(87)**	2009	Addicted prisoners (Sari)	312	30.8%
**Khedmat *et al. *(88)**	2007	Blood donors	318029	0.09%
**Merat *et al. *(3)**	2009	General population	6583	0.5%

### 2.2. Hepatitis B and C in Europe

The prevalence of chronic HBV infection in the European general population ranges from 0.2% in Ireland and the Netherlands to over 7% in some parts of Turkey. The prevalence of HCV also varies from 0.4% in Sweden, Germany, and the Netherlands to over 2–3% in some Mediterranean countries (24, 25). The overall prevalence differs among racial and ethnic populations and is highest among individuals who emigrated from areas with high endemicity of HBV and HCV infections (e.g. Asia, Africa, and Middle East) (26, 27). The high-risk groups in Europe are the same as in Iran, i.e. those who have the possibility to contact with infected blood like drug users, prison-mates, and medical care personnel, but in addition, there is another high-risk group: immigrants from areas of high or intermediate prevalence of HBV and HCV infections (28). Meffre et al. found four main characteristics associated with high prevalence of both HBV and HCV infections in France that can also be applicable for other European countries: intravenous drug abuse, country of birth, low level of education, and lower socioeconomic level. These four characteristics can be considered as the key for designing the prevention, screening, and treatment systems (29). Within European region, most countries offer universal vaccination against HBV since 1991. However, the UK and Scandinavian countries still have advocated universal vaccination and focused on well-defined risk groups, due to economic bases (28). Surveillance data from Italy (where universal vaccination started in 1991 in infants as well as in adolescents) have shown a remarkable overall decline in the incidence of acute hepatitis B after vaccination implementation (30).

### 2.3. Hepatitis B and C in the USA

The prevalence of chronic HBV infection in the USA is 0.4-0.5%, and that of HCV is 1.8% (31). Chak et al. designed a study focused on the high-risk groups to estimate the prevalence of HCV infection among prisoners, homeless people, healthcare workers, injection drug abusers, persons on long-term dialysis, recipients of chronic blood transfusions (i.e. hemophiliacs), and non-active military personnel. This study revealed that there were at least 5.2 million persons living with HCV infection in the USA (32) (Table 2). Waslley et al. analyzed data from National Health and Nutrition Examination Surveys (NHANES) from 1988-1994 (NHANES 1988-1994) and 1999-2006 (NHANES 1999-2006); the prevalence of HBV infection decreased among persons 6-19 years old (from 1.9% to 0.6%) and 20-49 years old (from 5.9% to 4.6%) but not among persons > 50 years old (7.2% vs. 7.7%) (33). This reflects the impact on strategies to eliminate HBV transmission which started in 1991 in the USA including national vaccination program of infants, screening all pregnant women for HBV with post-exposure prophylaxis provided for infants born from infected women, catch up vaccination of adolescents, and vaccination of adults at increased risk of infection (34-36).

**Table 2:** Estimated Total Prevalence of Hepatitis C Virus in the USA among Variable Risk Groups

Risk Group	Prevalence Range, %
**Homeless people**	22.2 -52.5%
**Prisoners**	23.1 - 41.2%
**Non-active military personnel**	5.4 - 10.7%
**Healthcare workers**	0.9 - 3.6%
**Chronic haemodialysis**	7.8%
**Haemophiliacs with transfusions before 1992**	76.3 - 100%

### 2.4. Hepatitis B in Asia and Africa

Asia and Africa were classified as high endemic areas for hepatitis B infection, but due to the national vaccination programs, some countries became intermediate and low endemic areas (37). China is the only country in Asia that was classified as high endemic area with prevalence of 7-20% for HBV infection. Countries with intermediate endemicity include India, Korea, Taiwan, and Thailand, and those with low endemicity include Japan, Pakistan, Singapore, and Malaysia with 0.2-1.9% prevalence (38). Most countries in Africa are considered as high endemic areas with chronic HBV infection rates of 7-26% in West and East Africa. Central and Southern Africa is highly endemic regions except Zambia, which has borderline intermediate endemicity. North Africa is considered also as a highly endemic except Tunisia and Morocco, which have intermediate endemicity with infection rate below 7% (39). Most Asian countries have started hepatitis B prevention programs, but only few African countries have started infant vaccination programs. The vaccination program in some Asian countries has changed HBV endemicity. In Saudi Arabia, the prevalence of HBV infection in children decreased from 6.7% to 0.3% within eight years from starting the vaccination program (40). In Malaysia, HBV prevalence in 7-12 years old children decreased from 1.6% (in 1997) to 0.3% (in 2003) after the implementation of infant vaccination program in 1990 (41). Data about the efficacy of immunization programs in African countries are limited. A study from Egypt, which integrated HBV vaccine in the national immunization program, revealed that HBV prevalence among the studied group was 0% among children aged 2- 4 years, 2% among children aged 4 -13 years, and 6.66% among adults (42). El-Raziky et al. recommended mass screening for HBsAg of pregnant Egyptian women and giving a birth dose of HBV vaccine to decrease the vertical transmission of HBV infection (43).

### 2.5. Hepatitis C in Asia and Africa

Countries with the highest reported prevalence rates are located in Africa and Asia (44), with prevalence of 5.3% in Africa and 2.15-3.9% in Asia (45). The most common risk factors for HCV transmission are intravenous drug abuse and blood transfusion (44, 45). Contaminated injection instruments are the major risk factor of HCV infection. In Egypt, high HCV seroprevalence was attributed to contaminated glass syringes used in national wide Schistosomiasis treatment from 1960 to 1987 (46). Blood transfusions are also highly effective means of HCV infection transmission. WHO`s Global Database on Blood Safety estimated that 43% of donated blood in developing countries is not screened adequately for transfusion-transmitted infections, including HCV (47). There are other sources of HCV transmission such as hemodialysis and sexual transmission. The latter is not confirmed and the role of sexual activity in HCV transmission remains unclear (45); however, in a study among spouses in Egypt, 6% were estimated to have contracted HCV from their spouse (48).

### 2.6. HCC in Europe

There is a wide geographical variation in the incidence of HCC throughout Europe (49). The highest incidence reported in Southern Europe (12/100,000 in men and 3/100,000 in women), while the lowest incidence was in the North of Europe (3/100,000 in men and 1/100,000 in women). This increased incidence in Southern Europe is likely related to the past increase of risk factors (HBV infection, HCV infection, and alcohol intake) (5, 50). Hepatitis B and C infections and liver cirrhosis, either subsequent to hepatitis or alcohol-related, are the classic risk factors for HCC, but associations with smoking, aflatoxins, and oral contraceptives also have been identified (51, 52). About 80% of HCC patients are infected with HBV or HCV infections (53). HBV infection increases the incidence of developing HCC by hundred times compared to those who are not infected (54). Cirrhotic patients have a higher risk of HCC with annual incidence of 2-6.6 %, whereas it is 0.4% in non-cirrhotic patients (55). Heavy (> 50-70 g/d) and persistent alcohol consumption (leading to cirrhosis and alcoholic hepatitis) increase the risk of HCC. The risk of cirrhosis and HCC are further increased in heavy drinkers who have HBV and/or HCV infections (56). Dietary ingestion of aflatoxins (produced by the mould Aspergillus flavus, which contaminates stored grains under hot and humid settings) is causally associated with the development of HCC, and exposure to aflatoxins may be synergistic with HBV infection (57). Hepatitis D virus (HDV) superinfection in patients with chronic hepatitis B infection leads to early cirrhosis and decompensation. It may be speculated that hepatocellular carcinoma differs in these patients from hepatitis B virus (HBV) monoinfection (58). HDV infection is much more common in the immediate Mediterranean region, sub-Saharan Africa, the Middle East, and the northern part of South America. In all, about 20 million people may be infected with HDV (59, 60).

### 2.7. HCC in the USA

The incidence of HCC in the United States has historically been lower than that in other countries (61). The incidence has doubled from 1.6 per 100,000 (1975-1980) to 3 per 100,000 (2001-2006) (9, 62). The increased incidence of HCC in the United States may be related to the late complications of HCV infection, which increased considerably in 1960s and reached to its peak in 1980s (63, 64). Due to the long period between HCV infection and cirrhosis, further increase in the number of HCC is expected to occur (63, 65). This is magnified also by improved medical care of cirrhosis, which gives more time for HCC to develop (66). The national immunization program for HBV vaccine in the USA contributed to the decrease in HBV infection as a risk factor for HCC (67). Yang et al. stated that most patients diagnosed having HCC from 1976 to 2000 in Minnesota/USA had alcohol-related liver disease as a risk factor, but from 2001 to 2008, HCV was the most common identified risk factor (68). HCC incidence in the USA was highest among Asian/Pacific Islanders (7.8 per 100, 000) followed by blacks (4.2 per 100, 000), American Indians (3.2 per 100, 000), and whites (2.6 per 100, 000). The high prevalence among Asians and African living in the USA can be attributed to their origin from endemic areas HBV and HCV infections (62).

### 2.8. HCC in Asia

HCC's are considered as one of the most frequent malignancies in Asia. The average prevalence ranges from 14-36 per 100, 000 persons with the highest prevalence in China (26-32 per 100,000 persons) (6) that reaches to 70-80 per 100,000 persons in the east coastal areas of China (69). There is a difference in the incidence rate between different countries in Asia, and between male and female with male to female ratio of 3:1. More than 70% of all newly discovered HCC occur in Asia, which about 75% of those are infected with HBV. China alone accounts for 55% of HCC cases in the world (70, 71). The incidence of HCC remains static over the last 20 years in most of Asian countries except of Singapore, where the incidence decreased markedly, especially in men, from 17 per 100,000 persons between 1968-1972 to 7.1 per 100,000 persons between 1998-2002 (72). On the other hand, China and Taiwan had reported a considerable increase in the incidence of HCC among both men and women. This can be attributed to the increased awareness and better screening and diagnosis services (69). The main etiology agent of HCC in Asia is HBV infection, which usually accounts for 70% of cases (73). In China, HBV was detected in 67% of patients, whereas HCV in 4 %. In Japan, HBV was only detected in 18% and HCV in 70% of patients. In Singapore, 35% of patients were infected with HBV and 13% with HCV (72-74). A survey about HCC in Asian countries showed that about 20% of patients from China and Indonesia and about 10% of patients in Japan were negative to both HBV and HCV infections; the etiology was unknown.

### 2.9. HCC in Africa

There is a wide regional variation in the incidence of HCC in Africa; Middle, Eastern, and Western Africa are considered as high incidence areas (15.3-27.8 per 100,000 persons in males and 5.6-13.4 per 100,000 in females) whereas Southern and sub-Saharan Africa are considered as intermediate incidence areas for HCC (7 per 100,000 persons in males and 2.5 per 100,000 in females). Northern Africa (except Egypt) is considered as a low incidence area (4.2 per 100,000 persons in males and 2.2 per 100,000 persons in females) (71). Egypt is considered as a high incidence area of HCC (16.4 per 100,000 for males and 4 per 100,000 in females) (75). There are limited studies about the progression of HBV and HCV infections in Africa. HCC distribution in Africa follows those seen in distribution of HBV and HCV infections. A study from Gambia reported that HBV infection, HCV infection, and aflatoxin exposure were well-documented etiologies of HCC, with HBV infection accounting for the majority of cases. Alcohol played a minor role as a risk factor for HCC (76-78). Another study about HCC development in North Africa in 2011 revealed that 60% of HCC patients in the study were infected with HCV and only 17.9% with HBV (79). These results were different from that were reported by another group in North Africa in the 1990s in which the authors revealed that HBV prevalence among HCC patients was 60% (80). This can be attributed to the widespread introduction and efficacy of HBV vaccine in this region. In Egypt, the high HCC prevalence can be attributed to the endemicity of HCV infection, which reaches to 14%. Some studies reported HCC patients without clearly identified etiological agents. This can be explained by development of occult viral infection, especially HBV, with low HBV-DNA concentration in serum being detected by conventional serological assays (78, 81).

### 2.10. HCC in Iran

Although the true prevalence of HCC in Iran is unknown, it is considered as a low risk area for HCC with the incidence less than five per 100,000 populations (82). In contrast to the western countries, alcohol consumption plays a minor role for HCC development in Iran. A study on the risk factors of HCC in southern Iran revealed that only 2.8% of HCC patients had a history of excess alcohol intake. The same study showed that the predominant cause of HCC in the studied group was hepatitis B followed by hepatitis C infections with an incidence of 52.1% and 8.5%, respectively (83). About 80% of HCC patients were positive for at least one of the known hepatitis B markers. Thus, HBV infection appears to be the most common cause of HCC in Iran (17). Alavian et al. reported about 1.5 million people in Iran as infected with hepatitis B and 15-40% of them with a possibility to develop cirrhosis and/or hepatocellular carcinoma (84).

## 3. Results

### 3.1. Worldwide

**Figure 1:**
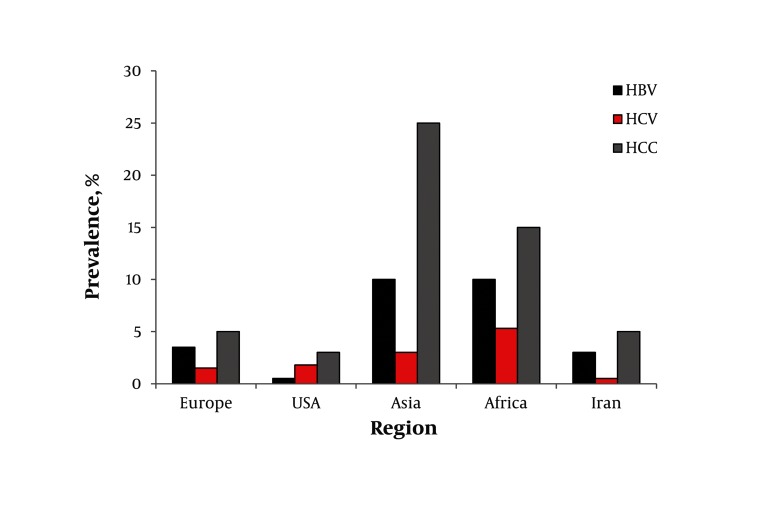
Distribution of Hepatitis B Virus (HBV) Infection, Hepatitis C Virus (HCV) Infection, and Hepatocellular Carcinoma (HCC) in Iran and all Over the World

Hepatitis B and C virus infections considered as major health threats, and their distributions among the world differ widely, with the highest incidence in Asia and Africa. The distribution of hepatocellular carcinoma follows that of HBV and HCV infections all over the world. It intensifies the fact that HBV and HCV infections are considered as the main risk factors of HCC (Figure 1). Alcohol as a risk factor for HCC plays a minor role in Asia and Africa, in spite of its noticeable role in Europe and the USA. After introduction of vaccination programs in most countries all over the world, there is a gradual decline in the prevalence of HBV infection in many countries with consequence decrease in HBV-related HCC.

### 3.2. Iran

Iran is considered as a low endemic area for hepatitis B and C infections, as well as for HCC (< 5 per 100,000 persons). Hepatitis B and C infections are the main causes of HCC in Iran, and prevention and treatment of these infections are the cornerstones to prevent HCC. There is no significant difference shown in the prevalence of HBV before and after the introduction of the national vaccination program in 1992, with an expected decrease in the future. There is a relatively high prevalence of HCV infection among risk groups in Iran. This may increase the burden on the health system and magnify the role of HCV infection as a risk factor for HCC in Iran, especially after the decline of HBV infection in the future.

## 4. Conclusions

### 4.1. Worldwide

- Vaccination remains the most effective preventive approach against HBV.

- Vaccination programs should be extended to include those who are at risk for HBV infection with screening of the pregnant women to identify those who are infected with HBV, and to give their infants a booster dose on birth.

- More screening programs should be directed to risk groups of Hepatitis B and C infections.

- Improving the awareness of the threat posed by HBV and HCV infections, the public health systems need to increase the awareness about modes of transmission, risk groups, consequences of HBV and HCV infections, and their relations to cirrhosis and HCC among the population.

- Although the incidence of HCC continues to rise, it is expected that vaccination programs will reduce the rate of HBV-related HCC in the next years.

### 4.2. Iran

- Although there is no clear difference in the epidemiology of HBV in Iran before and after introduction of the national vaccination program in 1992, a significant decrease in the prevalence is expected in the next years.

- After the decrease of HBV prevalence, HCV infection will replace HBV infection as major cause of cirrhosis and HCC.

- HCV could be completely eradicated by primary prevention through increasing the awareness of population about the risk groups, modes of transmission, and HCC as a possible consequence to HCV infection.
